# Volume-outcome revisited: The effect of hospital and surgeon volumes on multiple outcome measures in oesophago-gastric cancer surgery

**DOI:** 10.1371/journal.pone.0183955

**Published:** 2017-10-26

**Authors:** Claudia Fischer, Hester Lingsma, Niek Klazinga, Richard Hardwick, David Cromwell, Ewout Steyerberg, Oliver Groene

**Affiliations:** 1 Erasmus MC, Department of Public Health, Rotterdam, The Netherlands; 2 Amsterdam Medical Center, Department of Public Health, Amsterdam, The Netherlands; 3 Cambridge Oesophago-Gastric Centre, Addenbrooke’s Hospital, Cambridge University Hospitals NHS Foundation Trust, Cambridge, United Kingdom; 4 Department of Health Services Research and Policy, London School of Hygiene & Tropical Medicine, London, United Kingdom; 5 OptiMedis AG, Hamburg, Germany; Northwestern University, UNITED STATES

## Abstract

**Background:**

Most studies showing a volume outcome effect in resection surgery for oesophago-gastric cancer were conducted before the centralisation of clinical services. This study evaluated the relation between hospital- and surgeon volume and different risk-adjusted outcomes after oesophago-gastric (OG) cancer surgery in England between 2011 and 2013.

**Methods:**

In data from the National Oesophago-Gastric Cancer Audit from the UK, multivariable random-effects logistic regression models were used to quantify the effect of surgeon and hospital volume on three outcomes: 30-day and 90-day mortality and anastomotic leakage. The models included patient risk factors to adjust for differences in case-mix among hospitals and surgeons. The between-cluster heterogeneity was estimated with the median odds ratio (MOR).

**Results:**

The study included patients treated at 42 hospitals and 329 surgeons. The median (interquartile range) of the annual hospital and surgeon volumes were 110 patients (82 to 137) and 13 patients (8 to 19), respectively. The overall rates for 30-day and 90-day mortality were 2.3% and 4.4% respectively, and the anastomotic leakage was 6.3%. Higher hospital volume was associated with lower 30-day mortality (OR: 0.94; 95% CI: 0.91–0.98) and lower anastomotic leakage rates (OR: 0.96; 95% CI: 0.93–0.98) but not 90-day mortality. Higher surgeon volume was only associated with lower anastomotic leakage rates (OR: 0.81; 95% CI: 0.72–0.92). Hospital volume explained a part of the between-hospital variation in 30-day mortality whereas surgeon volume explained part of the between-hospital variation in anastomotic leakage.

**Conclusions:**

In the setting of centralized O-G cancer surgery in England, we could still observe an effect of volume on short-term outcomes. However, the effect is inconsistent, depending on the type of outcome measure under consideration, and much smaller than in previous studies. Efforts to centralise O-G cancer services further should carefully address the effects of both hospital and surgeon volume on the range of outcome measures that are relevant to patients.

## Introduction

For many surgical procedures, patient outcomes have been found to be related to surgical volume (the number of procedures that is performed in a specific unit), with studies typically showing that higher volumes are associated with lower postoperative mortality [1]. As a result, the centralization of high-risk oncological services, including oesophago-gastric cancer (O-G cancer), is occurring in many countries [2–6]. In the UK, the Department of Health published a recommendation to centralize curative surgical services into specialised cancer centres in 2001 [7]- and it is recommended that surgeons perform a minimum of 15–20 annual resections[8]. As a consequence, a process of reorganization has taken place in the National Health Services (NHS) during the past decade, which has resulted in a smaller number of acute trusts (hospital organisations) doing this type of surgery.

In O-G cancer surgery, risk-adjusted postoperative mortality and complication rates are widely used as quality indicators[9]. Case volume has also been proposed as a marker for quality in the past because of the substantial evidence of a volume-outcome relationship[10]. However, it is unclear whether a volume-outcome relationship is still detectable given that, in a centralized setting, all trusts may exceed recommended thresholds. In addition, the exact mechanism behind the volume-outcome relation is still not fully understood[11,12]. It is suggested that both the experience of the surgeon and the complete hospital team contribute to surgical outcomes[10]. Finally, there are more outcomes of interest for O-G cancer surgery than the commonly used 30-day mortality. Recent publications suggest that anastomotic leakage rates and 90-day postoperative mortality are also important in assessing the quality of surgical care [13–17].

This study was undertaken to examine the relation between hospital- and surgeon volume and different risk-adjusted outcomes after O-G cancer surgery in a setting of centralized care, and the between-hospital and surgeon differences in outcome.

## Methods

### Data

We used data submitted to the National Oesophago-Gastric Cancer Audit (NOGCA), which evaluates the care delivered by all (n = 154) English hospitals that provide care to adults diagnosed with invasive, epithelial cancer of the oesophagus or stomach. Data are collected prospectively by hospital staff and have been submitted to the audit since 1 April 2011. Details on the audit method and dataset have previously been published[18]. All patients undergoing curative surgery between 1 April 2011and 31 March 2013 were included in the study. The date of death was obtained from the Office for National Statistics Death Register. We excluded patient undergoing curative oncological treatment for squamous cell carcinoma and all palliative patients. Further, we excluded hospitals which operated on less than 10 patients (S1 Fig).

### Predictors and outcomes

We considered three outcomes: 30-day mortality, 90-day mortality and anastomotic leakage. Mortality was defined as all-cause postoperative mortality within 30 or 90 days after surgery. Anastomotic leakage was defined as severe disruption to the anastomosis, irrespective of whether detected clinically or radiologically, and irrespective of whether it is managed conservatively or by re-operation[19]

Pre-operative patient and tumour characteristics to be used for case-mix adjustment were based on prior research. All regression models included: comorbidity count, age, ASA score, ECOG (WHO) performance status, T stage, N stage, cancer location. Patient gender was also included in the models for the outcomes 90-day mortality and AL, and deprivation was included in the AL model. We analysed hospital and surgeon volume. Hospital was defined as NHS trust, which is a division within the English NHS that can consist of up of up to five hospitals. Usually O-G cancer surgery is only performed in one hospital in the trust. Surgeon was defined as the principal operating surgeon.

Hospital volume was defined as the number of O-G cancer surgeries performed at a NHS trust per year. Surgeon volume was defined as the annual number of operations conducted by an individual surgeon.

### Statistical analysis

Patient characteristics were described as means or percentages. We described surgeon and hospital volume in the study period with median volume per hospital/surgeon. To describe outcome differences, we divided the hospitals/surgeons in quartiles based on volume and presented the outcome rates for each volume quartile.

The effect of hospital and surgeon volume on the three patient outcomes was tested in multivariable case-mix adjusted logistic regression models, with volume added as a continuous variable. We tested whether the relationship between volume and the outcomes was non-linear by adding squared terms and comparing these with linear terms based on the chi-square statistic.

We assessed differences in outcome between hospitals with random effects models[20]. First, we analysed between hospitals and surgeons differences without any adjustment. This random effects model includes two random intercepts: one for hospital and one for surgeon and no other covariates. The variance of the random intercepts represents the between hospital/surgeon variation without any adjustment, but taking into account the random variation. In the second model the case-mix adjustment variables were added as covariates, to adjust the between hospital/surgeon variation for differences in patient characteristics. In a next step, we added surgeon and hospital volume one by one as covariates. In a final step, we included both surgeon and hospital volume.

In random effects models the between hospital/surgeon variation is reflected in the variance of the random intercepts (τ2). We used the median odds ratio (MOR) to quantify this variation. The MOR is a direct function of τ2 (MOR = exp (√ 2 x τ2 x Φ^–1^ (0.75)) ^21^. The MOR can be equal or greater than 1, an MOR of 1 reflects no variation between the hospitals. The larger the between-hospital variation, the higher the MOR will be[21].

There were no missing values in patient outcomes. Missing data in predictors were imputed using a ‘multiple imputation by chained equations’ model including the outcome measures and relevant covariates. Imputation and statistical analysis were performed Stata software, version 11(StataCorp.2009.Stata Statistical Software: Release: 11) and in R statistical software 2.7.2 using the Hmisc, the lm4 and rms packages (R Foundation for Statistical Computation, Vienna) Syntax code for R is provided in the appendix (S1 Text).

## Results

### Descriptives

The study included 4868 patients treated at 42 hospitals and 329 surgeons. Patients had a mean age of 66 years and the majority were male (n = 3610; 74%). See Table 1 for all patient characteristics. Overall, 30-day mortality was 2.3% (n = 111), 90-day mortality was 4.4% (n = 215), and 6.3% (n = 305) of the patients developed an anastomotic leakage (AL).

**Table 1:** Characteristics of the patients included in the study.

Patient and prognostic information	No. of patients	%
**Age, years**	4859	66*
Missing values	9	0.2
**Comorbidity count**		
No comorbidities	2735	56.2
One comorbidity	1309	26.9
Two comorbidities	566	11.6
Three or more comorbidities	258	5.3
**Gender**		
Male	3610	74.2
**ECOG (WHO) performance status**		
Carries out all normal activity	2515	51.7
Restricted but walks/does light work	1550	31.8
Walks, full self-care but no work	526	10.8
Limited self-care—fully disabled	120	2.5
Missing values	157	3.2
**Size and/or extent of the primary tumour (T)**		
No evidence of primary tumour T(0)	202	4.1
Tumour invades lamina propria or submucosa T(1)	927	19.0
Tumour invades muscularis propria T(2)	790	16.2
Tumour invades adventitia T(3)	2318	47.6
Tumour invades adjacent structures T(4)	489	10.0
Missing values	142	2.9
**Regional lymph nodes (N)**		
No regional lymph node metastasis N(0)	2137	43.9
Metastasis in 1 to 2 regional lymph nodes N(1)	1496	30.7
Metastasis in 3 to 6 N(2)	614	12.6
Metastasis in 7 or more N(3)	507	10.4
Missing values	114	2.3
**ASA Scale**		
Normal healthy patient	811	16.7
Mild systemic disease	2498	51.3
Severe systemic disease	1246	25.6
Life-threatening disease/ Moribund patient	60	1.2
Missing values	253	5.2
**Cancer location**		
Squamous cell carcinomas of the oesophagus	491	10.1
Adenocarcinomas of the upper and middle oesophagus	183	3.8
Adenocarcinomas of the lower third of the oesophagus and Siewert type 1 tumours	1904	39.1
Siewert type II and type III tumours	840	17.3
Tumours of the stomach	1450	29.8
**Deprivation**		
1 Least deprived	838	17.2
2	859	17.6
3	844	17.3
4	799	16.4
5 Most deprived	745	15.3
Missing values	783	16.1

*Mean

#### Variation on surgeon and hospital level

**Fig 1:**
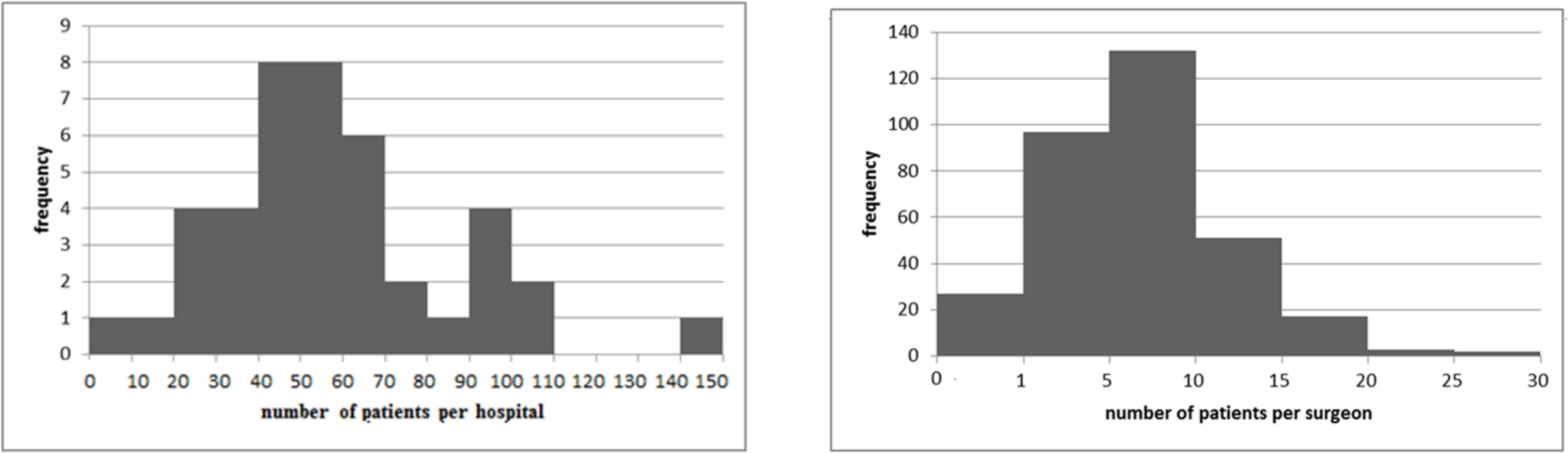
**ab. Hospital volume (1a), surgeon volume (1b).** (1a). Median hospital volume = 55; Interquartile range = 41–68. (1b). Median surgeon volume = 6; Interquartile range = 4–9.

Volume varied between both surgeons and hospitals. The median hospital volume was 55 patients per year, with an interquartile range of 41 and 68 (Fig 1). The median surgeon operated 6 patients per year, and the interquartile range was 4 and 9 (Fig 1).

The risk of all outcomes was lower in the highest volume quartiles compared to the lowest volume quartiles (Table 2). For example, 30-day mortality was 3.0% in the lowest hospital volume quartile compared 1.3% in the highest quartile.

**Table 2:** 30-day, 90-day mortality and anastomotic leakage risk according to quartiles of hospital volume and surgeon volume.

		Average hospital volume				Average surgeon volume			
	Total	Q 1	Q 2	Q 3	Q 4	Q 1	Q 2	Q 3	Q 4
		(0–49)	(50–65)	(66–91)	(92–148)	(0–5)	(6–9)	(10–13)	(14–28)
N		1,253	1,148	1,360	1,107	1,144	1,156	1,292	1,169
30-day mortality, %	2.3	3.0	3.1	1.7	1.3	2.4	2.3	2.6	0.7
90-day mortality, %	4.4	5.0	5.0	3.8	3.9	4.5	5.4	4.0	1.4
Anastomotic leakage,%	6.3	7.1	8.9	6.3	2.5	7.9	7.1	4.6	1.3

Higher hospital volume was a significant independent predictor for lower 30-day mortality (OR: 0.94 (95%CI: 0.91–0.98) per increase of 5 patients per year) (Table 3). Surgeon volume had no significant effect on 30-day mortality. Higher surgeon and higher hospital volume were independent predictors of lower risk of an anastomotic leakage (hospital volume OR: 0.96 (95%CI: 0.93–0.98), surgeon volume: OR: 0.88 (95% CI: 0.78–1.00). Neither hospital volume nor surgeon volume were significant predictors for 90-day mortality when controlling for each other (Table 3).

**Table 3:** Association between adjusted volume predictors and the outcomes 30-day mortality, 90-day mortality and anastomotic leakage.

	30-day mortality		90-day mortality		Anastomotic leakage	
	OR	95%CI	OR	95%CI	OR	95%CI
Surgeon volume*						
Adjusted for case-mix	1.03	0.84–1.25	1.01	0.87–1.16	**0.88**	**0.78–1.00**
Adjusted for case-mix and hospital volume	0.92	0.76–1.12	0.97	0.85–1.11	**0.81**	**0.72–0.92**
Hospital volume*						
Adjusted for case-mix	**0.94**	**0.91–0.98**	0.98	0.96–1.00	**0.95**	**0.93–0.97**
Adjusted for case-mix and surgeon volume	**0.94**	**0.91–0.98**	0.98	0.96–1.01	**0.96**	**0.93–0.98**

* ORs represent the effect of 5 extra patients per year

**Fig 2:**
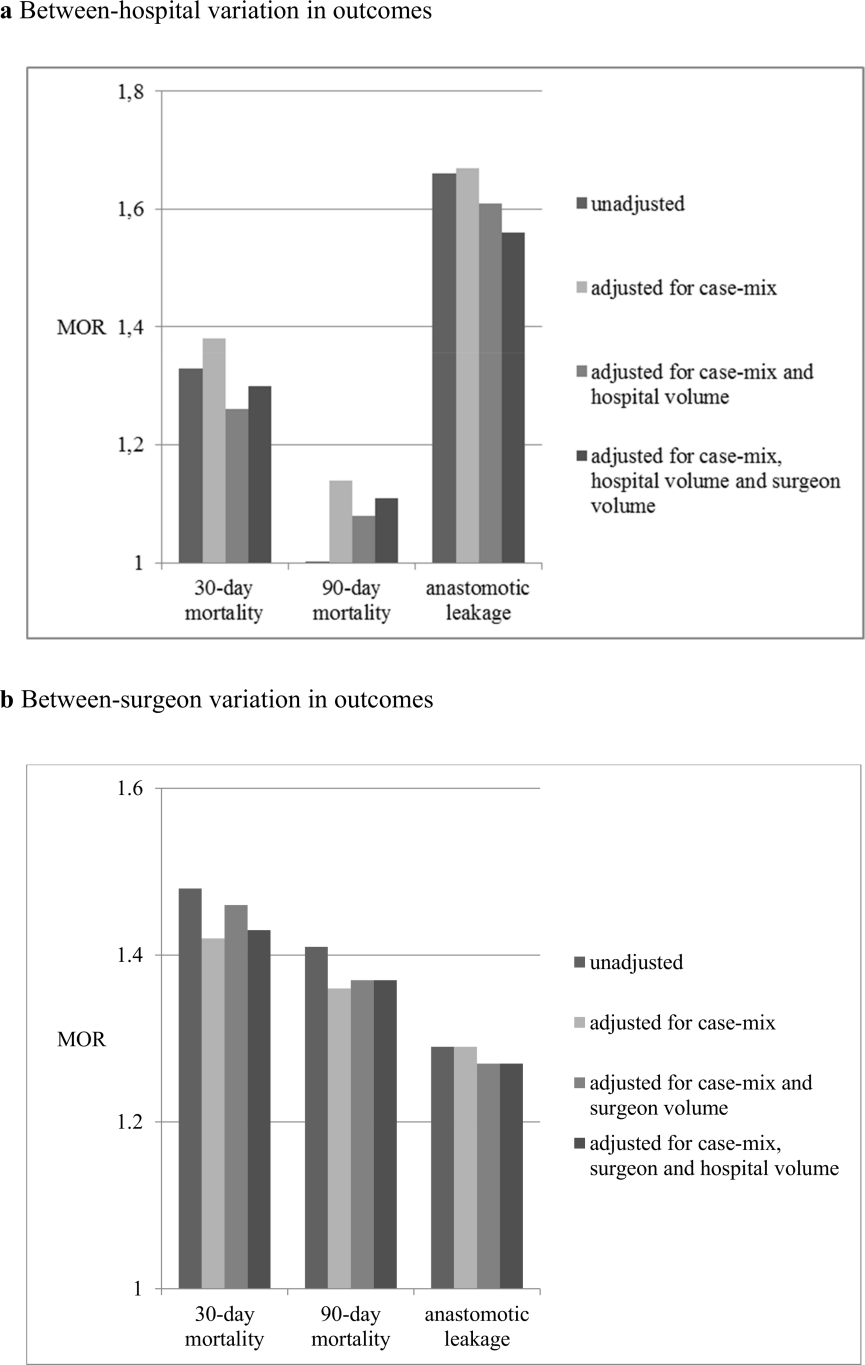
Change in median odds ratio (MOR) on surgeon level considering different case mix and prediction factors for 30-day, 90-day mortality and anastomotic leakage. **Fig 2 a. Between-hospital variation in outcomes.** Change in median odds ratio (MOR) on hospital level considering different case mix and prediction factors for 30-day, 90-day mortality and anastomotic leakage. **Fig 2 b. Between-surgeon variation in outcomes.** Change in median odds ratio (MOR) on surgeon level considering different case mix and prediction factors for 30-day, 90-day mortality and anastomotic leakage.

Hospital volume explained part of the variation in 30-day mortality between the hospitals. The median odds ratio (MOR) decreased from 1.38 without controlling for hospital volume in the model to 1.30 when hospital and surgeon volume were added to the models as covariates (Fig 2). The MOR represents the odds of dead within 30 days of a patient from a randomly selected hospital compared to the odds if he/she would go to another randomly selected hospital, after taking into account the random variation and differences in patient characteristics. Surgeon volume did not explain between-hospital or between-surgeon variation in 30-day mortality (Fig 2, S1 Table).

Hospital volume explained more of the between-surgeon variation in anastomotic leakages (change in MOR from 1.67 to 1.56) (Fig 2). The other way around, surgeon volume explained a very small part of the variation between hospitals (change in MOR from 1.29 to MOR 1.27) (Fig 2).

While hospital volume explained a minimal amount of between-hospitals variation in 90-day mortality, surgeon volume did not explain any variation in 90-day mortality at all (Fig 2).

## Discussion

### Main findings

This study has shown that despite centralization, differences between hospitals and surgeons in patient outcomes after O-G surgery still exist in England. These between-hospital and between-surgeon differences are partly explained by surgeon and hospital volume but the volume outcome relation is different for the different outcomes (30-day and 90-day mortality and anastomotic leakage). Higher hospital volume was associated with lower 30-day mortality, but high surgeon volume was not. Neither hospital volume nor surgeon volume affected 90-day mortality. Higher surgeon and hospital volume were both associated with fewer anastomotic leakages, but surgeon volume was the stronger predictor.

#### Volume-outcome relation

Higher volume hospitals have lower 30-day mortality rates; this in line with earlier research in both O-G cancer and other surgical oncological procedures [3,22].

Despite the centralization that took place in the UK, we still found an effect of volume.

In contrast, we did not find surgeon volume to be a significant predictor of 30-day mortality. Surgeon volume and hospital volume possibly reflect different aspects of quality of care. It has been suggested that next to the surgeon skills there are other hospital factors in high volume hospitals that reduce mortality risk, such as post-operative care, care pathways and multidisciplinary team work [23–26]

Surgical volume and hospital volume had only a minor effect on 90-day mortality. Although still a substantial amount of patients dies between 30- and 90-days after surgery, their death seems not to be influenced by the surgeon or hospital volume. Previous research showed that although part of the deaths occurring after 30 days is still related to surgery, and increasing proportion related to cancer recurrence [14]. Both, higher surgeon and hospital volume showed to be related to fewer anastomotic leakages, but surgeon volume had a stronger effect. It can be imagined that anastomotic leakages are closely related to technical surgical skills that high volume surgeons have better developed.

When calculating surgeon volume for highly complicated but rare procedures, it has been suggested to count also related procedures[27]. In our study we had no data to take into account other operations as O-G resections.

#### Recommendations

In different countries different volume norms are used, which is the reflection of mixed scientific evidence. Our findings suggest that further increasing hospital volumes, and to a lesser extent surgeon volumes, might improve short-term outcomes. However, on-going centralization might also have negative effects on for example access and equity, which we did not study.

We studied different outcomes that are used as quality indicators. Anastomotic leakages (AL) rates were affected by hospital volume. This is in line with previous findings that surgical complications seem to be a good indicator for surgical quality as they are closely related to the surgical process and are not so much influenced by patient characteristics[9,28,29]. This makes AL rates ‘actionable’ hospital quality indicators. Apart from the clinical relevance, the relatively frequent occurrence makes them attractive as a quality indicator from a statistical point of view, as rates per hospital can be estimated relatively certain. A large disadvantage of complications as an outcome indicator is their possibly unclear definition, which may bias between hospital comparison[30].

Adjusted 30-day mortality differences between hospitals were partly explained by hospital volume. However, a large disadvantage of this measure is the low event rate. Even in our data pooled from two years, the absolute numbers of deaths within 30-days per hospital were small (median absolute number per hospital = 2), which makes the estimates per hospital uncertain and challenges the comparison between hospitals [31,32].

In that sense 90-day mortality rate is more attractive as more deaths occur. However, 90-day mortality differences between hospitals were not explained by hospital volume. Possibly the 90-day mortality rates might reflect aspects of quality of care not related to volume. But we consider it is more likely that a longer time period introduces effects of confounding factors that dilute the relation between quality of care and outcome. In all, multiple indicators should be monitored as this gives a more comprehensive picture [33].

Next to variation between hospitals, we did observe variation in outcome between surgeons. But these were not explained by any of the volume indicators. In addition, the absolute numbers of deaths of ALs per surgeon are extremely low, which makes outcome rates per surgeon unsuitable as quality indicators: the difference between 0 or 1 death per year cannot tell us anything about quality, only about bad luck.

In summary, in the setting of centralized O-G cancer surgery in England, we could still observe an effect of hospitals volume on 30-day mortality and AL rates, suggesting that further centralization might be considered but should carefully address the effects of both hospital and surgeon volume on the range of outcome measures that are relevant to patients. AL rates and 30-day mortality rates per hospital could be useful as quality indicators, but both have also disadvantages. 90-day mortality likely reflects other things than the quality of surgery and thus is less suitable as a quality indicator. Surgeon level outcomes are so infrequent that they are not suitable as quality indicators.

## Supporting information

S1 Fig Flow chart patient inclusion process. (DOCX)

S1 Text R code. (DOCX)

S1 Table Between surgeon and between-hospital variation in 30-day, 90-day mortality and anastomotic leakage. *Median odds ratio (95% Confidence Interval).
(DOCX)
