# Supplementary material for: Volume-outcome revisited: The effect of hospital and surgeon volumes on multiple outcome measures in oesophago-gastric cancer surgery
Source: PLoS One. 2017 Oct 26;12(10):e0183955. doi: 10.1371/journal.pone.0183955 (PMC5658198; doi:10.1371/journal.pone.0183955)
Supplement: S1 Fig — (DOCX) [file pone.0183955.s001.docx]

**S1_Fig. Flow chart patient inclusion process.**

Patients with primary diagnosis of OG cancer between 1.4.2011 and 31.3.2013

n=22,766

Excluded patients without surgical treatment plan (either oncology or endoscopic treatment only or best supportive care)

n= 17403

Patients undergoing oesophagectomy or gastrectomy = 5567

Excluded patients with palliative surgical intent (n=377) and non-curative procedure (open-and-shut or bypass procedure, n=217)

n= 594

Patients with curative oesphago-gastric resection

n=4973

Excluded records with missing or incorrect consultant GMC codes

n=91

Patients with curative oesphago-gastric resection included in the analysis

n=4882

Excluded hospitals, which operated on less than 10 patients (n = 10)

n= 14

Patients with curative oesphago-gastric resection included in the analysis

n=4868
