# Supplementary material for: Volume-outcome revisited: The effect of hospital and surgeon volumes on multiple outcome measures in oesophago-gastric cancer surgery
Source: PLoS One. 2017 Oct 26;12(10):e0183955. doi: 10.1371/journal.pone.0183955 (PMC5658198; doi:10.1371/journal.pone.0183955)
Supplement: S1 Text — (DOCX) [file pone.0183955.s002.docx]

**S1_Text.** **R code**.

**#Load required packages**

library(lm4) #fits random effect logistic regression models

library(foreign) #can import foreign data files

**#Import STATA datafile**

MyData <- read.dta(“S:/MyDocuments/……………..dta”, convert.dates=TRUE, convert.factors=TRUE, missing.type=TRUE, convert.underscore=TRUE)

**#Random effects model with 30-day mortality as outcome, patient characteristics, surgeon and #hospital volume as covariates, and two random effects: one for surgeon and one for hospital #(Figure 1, Figure 2, outcome 30-day mortality bar group, last bar)**

MyModel <- glmer(30day.mortality ~ volume.surgeon + volume.hospital + age + as.factor(comorbidity) + as.factor(performancestatus) + as.factor(t)+ as.factor(n) + as.factor(asa)+ as.factor(tumour.location) + (1| surgeon) + (1|hospital), data=MyData, family=binomial)

**#MOR between surgeons**

exp(0.674*(sqrt(as.numeric(VarCorr(MyModel)[[1]]))))

**#MOR between hospitals**

exp(0.674*(sqrt(as.numeric(VarCorr(MyModel)[[2]]))))
