# Supplementary material for: Volume-outcome revisited: The effect of hospital and surgeon volumes on multiple outcome measures in oesophago-gastric cancer surgery
Source: PLoS One. 2017 Oct 26;12(10):e0183955. doi: 10.1371/journal.pone.0183955 (PMC5658198; doi:10.1371/journal.pone.0183955)
Supplement: S1 Table — *Median odds ratio (95% Confidence Interval). (DOCX) [file pone.0183955.s003.docx]

**S1_Table. Between surgeon and between-hospital variation in 30-day, 90-day mortality and anastomotic leakage.**

|  | | **Unadjusted** | **Adjusted for patient characteristics** | **Adjusted for patient**  **characteristics and hospital volume** | **Adjusted for patient**  **characteristics and surgeon volume** | **Adjusted for patient**  **characteristics, surgeon and hospital volume** |
| --- | --- | --- | --- | --- | --- | --- |
|  |  | **MOR*** | **MOR** | **MOR** | **MOR** | **MOR** |
| **30-day mortality** | |  |  |  |  |  |
| Combined between surgeons | | 1.48  (1-2.93) | 1.42  (1-2.87) |  | 1.46  (1-2.91) | 1.43  (1-2.88) |
| Combined between hospitals | | 1.33  (1-1.81) | 1.38  (1 – 1.86) | 1.26  (1 – 1.74) |  | 1.30  (1 – 1.78) |
| **90-day mortality** | |  |  |  |  |  |
| Combined between surgeons | | 1.41  (1-1.81) | 1.36  (1 – 1.76) |  | 1.37  (1 – 1.77) | 1.37  (1 – 1.77) |
| Combined between hospitals | | 1  (1-1.55) | 1.14  (1 – 1.69) | 1.08  (1 – 1.63) |  | 1.11  (1 – 1.66) |
| **Anastomotic leakage** | |  |  |  |  |  |
| Combined between surgeons | | 1.29  (1-1.71) | 1.29  (1 – 1.71) |  | 1.27  (1 – 1.69) | 1.27  (1 – 1.69) |
| Combined between hospitals | | 1.66  (1.63-2.01) | 1.67  (1.64 – 2.02) | 1.61  (1.58 – 2.02) |  |  |

*Median odds ratio (95% Confidence Interval)
